# The mediating role of perceived social support: alexithymia and parental burnout in parents of children with autism spectrum disorder

**DOI:** 10.3389/fpsyg.2023.1139618

**Published:** 2023-06-09

**Authors:** Yuanting Lin, Yan Wang, Chunhui Lin, Qingnan Ni, Ruolin Jia, Yanling Chang, YuanPing Qi

**Affiliations:** ^1^Pediatric Rehabilitation Department, The Third Affiliated Hospital of Zhengzhou University, Zhengzhou, China; ^2^Pediatric Rehabilitation Department, Qinghai Women and Children’s Hospital, Xining, China

**Keywords:** parental burnout, autism spectrum disorder, alexithymia, perceived social support, gender

## Abstract

**Background:**

Parental burnout is a concept that reflects the emotional exhaustion and emotional distance of parents from children due to their inability to cope with the pressure of parenting. It has been confirmed that parents of autistic children are at higher risk for parental burnout. Additional research has suggested a relationship between parental burnout and parents’ personality traits. However, the relationship between alexithymia, an independent personality factor, with parental burnout is little to none.

**Objective:**

To look into the connection between parental burnout and alexithymia among parents of autistic children.

**Method:**

Three hundred and one parents were approached for recruitment and data were collected from 203 parents through a cross-sectional survey assessing parental burnout, alexithymia status, and perceived social support. Because the data is not normally distributed, Spearman’s rank correlation coefficient rho(p) was used to assess the correlation between the variables; and then using AMOS to analyze the mediating effects of perceived social support and the moderating effect of gender.

**Result:**

The result showed that (1) There is a negative association between alexithymia with parental burnout (*β* = 0.6, *p* < 0.01), while perceive social support was the negative predictor of alexithymia (*β* = −0.45, *p* < 0.01) and parental burnout (*β* = −0.26, *p* < 0.01); (2) perceive social support partially mediated the relationship between alexithymia and parental burnout of parents of autistic children, which can explain 16.3% of the total effect; (3) Gender plays a moderating role in the first half of the indirect effect of alexithymia on parental burnout, as evidenced by the significant difference in path coefficients between the male and female models (male: *β* = −0.10, *p* < 0.05; female: *β* = −0.60, *p* < *0.05*).

**Conclusion:**

Health professionals and policymakers should be aware of parental burnout among parents of autistic children in China and take early intervention steps. Furthermore, they should recognize the negative impact of alexithymia and the positive impact of social support when developing plans to alleviate parental burnout in children with autism, with a particular focus on mothers with alexithymia, who are more likely to experience low social support and burnout than fathers with alexithymia.

## 1. Introduction

Children’s growth and development is a long process. Neurodevelopment delays are a main long-term issue among children under 5 years (Naz et al., 2023). Appearing in early childhood, autism spectrum disorder (ASD) is a neurodevelopmental disorder that is principally characterized by abnormal social interaction, impaired communication, and repetitive patterns of behavior (American Psychiatric Association Diagnostic and Statistical Manual of Mental Disorders, 2013). Autistic children often require additional support, which creates extra difficulties and challenges for parents. For example, as well as managing the child’s daily routine, parents need to learn how to manage the behavior, sleeping, and eating problems of autistic children (Baraskewich et al., 2021; Brown et al., 2021; Family Quality of Life Perceived by Mothers of Children with ASD and ADHD—PubMed, n.d.). As a result, parents of autistic children are to be under great parenting pressure (Hayes and Watson, 2013; Foody et al., 2015; DesChamps et al., 2020; Impact of Respite Care Services Availability on Stress, n.d.). When parenting resources are insufficient to cope with stress, parents may experience parental burnout, which is characterized by extreme exhaustion related to childcare, emotional distance from children, loss of enjoyment and efficacy in the parenting role, and comparison between past and present parenting selves (Roskam et al., 2018).

Depending on the characteristic of parents, the incidence of parental burnout was 8–36% (Lindahl Norberg et al., 2014; Roskam et al., 2017; Suárez et al., 2022). Previous studies have shown that parents of autistic children will experience more parental burnout, especially when children have a combination of physical and mental disabilities (Gérain and Zech, 2018; Kütük et al., 2021). Chronic parental burnout can affect parents’ physical and mental health, such as increased cortisol, insomnia, overeating, and anxiety, which can decrease their work efficiency (Brianda et al., 2020; Evans et al., 2022; Hormozi et al., 2022; Exhausted Parents in Japan, n.d.). Some parents may even resort to alcohol or suicide to avoid parental responsibilities (Mikolajczak et al., 2019, 2021). This resistance to children can also result in terrible marital and parent–child relationships, with marital conflict, violence, and neglect towards children (Hubert and Aujoulat, 2018; Mikolajczak et al., 2018; Mikolajczak and Roskam, 2020), and ultimately have long-term negative effects on children (e.g., academic burnout, behavior problems; Yuan et al., 2022; Zhang et al., 2023; Chinese Mothers’ Parental Burnout and Adolescents’ Internalizing and Externalizing Problems, n.d.).

According to the Balance between Risks and Resources (BR^2^) theory, parental burnout is the result of an imbalance between risk factors and protective factors (Mikolajczak and Roskam, 2018). From this perspective, identifying parental risk and protective factors can provide the basis for better burnout prevention. Burnout is inextricably linked to gender, and female seems to be risk factors for burnout because they are often asked to contribute more to parenting activities (Vigouroux and Scola, 2018). In addition, personality traits explain most of the causes of burnout in parents. Neuroticism and perfectionism are risk factors for parental burnout, while agreeableness and conscientiousness are protective factors (Le Vigouroux et al., 2017). However, previous research on personality traits of parental burnout has been conducted from the basic personality dimensions (five-factor model) and has not considered the impact of more specific and detailed personality structures.

As a personality trait, alexithymia is used to describe individuals who are unable to recognize and express emotions (Sifneos, 1973). The prevalence of alexithymia in the general public is 7–10% (Franz et al., 2008). In some families with neurologically impaired children, it has been proved that parents are at a higher risk of developing alexithymia (Durukan et al., 2018; Temelturk et al., 2021). Peter also concluded that parents’ alexithymia is associated with repetitive stereotyped behaviors of autistic children (Szatmari et al., 2008). It has been proved that most people with alexithymia have a neurotic personality and are able to perceive more negative emotions (like depression and anxiety; Heshmati and Pellerone, 2019; Mensi et al., 2020; Stability of Alexithymia and Its Relationships with the “big Five” Factors, Temperament, Character, and Attachment Style—PubMed, n.d.). Based on the existing evidence, it seems to be reasonable to conclude that alexithymia is a risk factor for parental burnout. However, more research is needed to prove this hypothesis. Therefore, this study attempts to examine the relationship between alexithymia and parental burnout and to analyze its mechanism of action.

Perceived social support refers to the external support that an individual can perceive, which was obtained from family, friends, and colleagues (Dunst et al., 1986). Alexithymia can also negatively impact an individual’s perceived social support. Due to cognitive and emotional deficits, individuals with alexithymia are often unable to take full advantage of social support, which can lead to social isolation and difficulties in seeking help (Celik et al., 2022). Perceived social support, however, as the social support that individuals can perceive when in contact with friends, family, and college, is an important protective factor against parental burnout (Ibarra-Rovillard and Kuiper, 2011). Not only it can reduce parenting stress by providing specific parenting help, for example, information and money, but also can increase parental resilience in the face of pain and obstacles (Lin et al., 2022; Yan et al., 2022).

To date, there has been a little survey to investigate parental burnout of parents whose children with autism in China. It does not mean that the parental burnout of Chinese parents is not important. Chinese parents have the most expectation for their newborns (GESIS-Suche: International Social Survey Programme: Family and Changing Gender Roles III—ISSP, 2002). Therefore, it has unique significance to explore the parental burnout of autistic children in the Chinese parenting environment. Synthesizing the above theoretical and research findings, this study intends to test three hypotheses: (1) alexithymia positively affects parenting burnout; (2) alexithymia can affect parenting burnout through perceived social support; (3) Gender may moderate the relationship between the three.

## 2. Methods

### 2.1. Participants

This study was conducted at the Pediatric Health Department of two tertiary hospitals in Zhengzhou City, between June 2022 and September 2022. A purposive sampling strategy and snowball sampling methods were used to recruit eligible parents of autistic children. The criteria for inclusion were as follows: (a) parents had one or more children that were diagnosed with autism spectrum disorder by a qualified doctor (meeting DSM-IV-TR diagnostic criteria); (b) possess Chinese reading ability; (c) Parents were excluded if they had a serious physical illness (including heart disease, malignant tumors, stroke, etc.) or a mental illness that might affect their participation (including autism spectrum disorder, schizophrenia, depression, anxiety, etc.).

### 2.2. Procedures

Behavioral and developmental physicians and researchers worked together to distribute recruitment flyers to parents whose children with ASD were undergoing rehabilitation in the hospital. The researcher would explain the research process to interested parents, and for the convenience of snowball sampling, we chose two methods for the study: on-site and online surveys. Specifically, after parents provided written informed consent, they were asked to complete a paper questionnaire survey to assess the status of alexithymia, perceived social support and parental burnout. If they were willing, they would be asked to refer other eligible participants to take part in the online survey. The initial recruitment sample consisted of 301 parents. After excluding all data with missing structural dimensions, we ended up with 203 valid responses, including 136 mothers and 67 fathers.

### 2.3. Measure

#### 2.3.1. Parental burnout

Roskam used the generalization method to form the Parental Burnout Assessment scale (PBA), which has been subsequently adopted by scholars and introduced in other countries (Roskam et al., 2018; Szczygieł et al., 2020). This study used the Chinese version of the Parenting Burnout Assessment scale to evaluate the parental burnout of parents with autistic children (Cheng et al., 2020). The Chinese version of the assessment scale is a single-factor structural scale consisting of 21 items. Parents rated the occurrence within each symptom on a seven-point Likert scale (1 = never, 7 = daily). The greater the parents’ score, the more burnout they experienced as parents. Cronbach’s alpha was 0.96 for the total score on this scale.

#### 2.3.2. Alexithymia

We used the 20-Item Toronto Alexithymia Scale (TAS-20) to evaluate the alexithymia of participants. TAS-20 is an effective measure of Alexithymia (Li et al., 2015). Consisting of 20 items, The TAS-20 assesses three components of Alexithymia: difficulty identifying feelings, difficulty describing feelings, and externally oriented thinking. Participants responded to the items according to the Likert 5-point system (1 = strongly disagree, 5 = strongly agree). The total scale is 20–100 points, with ≤ 51 points meaning non-alexithymia, 52–60 meaning borderline alexithymia status, and ≥ 61 classified as alexithymia. A higher score means more severe alexithymia (Bagby et al., 1994). Cronbach’s alpha was 0.85 for the total score on this scale.

#### 2.3.3. Perceived social support

We evaluated the informal support that participants experienced from friends, family, and other individuals using the Multidimensional Scale of Perceived Social Support (MSPSS). The MSPSS consisted of 12 items, which participants rated on a 7-point Likert scale (1 = very strongly disagree, 7 = very strongly agree). The total score was 12–84, and higher scores indicated greater perceptions of support (Zimet et al., 1988). Cronbach’s alpha was 0.91 for the total score on this scale.

### 2.4. Statistical analysis

Using IBM SPSS 26.0 and AMOS 24.0, we conducted and examined all of the data. For normally distributed data, we used arithmetic mean and standard deviation to describe; for not normally distributed data, we used median with percentile range to describe. Because the scores of parental burnout were non-normally distributed, the *Kruskal-Wallis H test* or *Mann–Whitney U test* was used to compare parental burnout with different demographic characteristics, and Spearman’s rank correlation coefficient rho*(p)* was used to assess the correlation between the variables (Bishara and Hittner, 2012). Finally, we used AMOS for mediating effects as well as moderating effects analysis.

## 3. Results

### 3.1. Characteristics of parental burnout of parents of autistic children

67% of the 203 valid responses received were from women, 62.6% of which were aged 30–40. The result shows that gender, monthly income (RMB)—and reimbursement method of treatment expenses were significantly related to parental burnout (*p* < 0.05). Specifically, women have a greater sense of parental burnout than men; The higher the monthly income, the lighter the sense of parental burnout; Among the reimbursement methods for rehabilitation treatment expenses, parents with commercial insurance have the least sense of burnout, all the data are shown in Table 1.

**Table 1:** Demographic characteristics of the study participants and the distribution of parental burnout.

Variable	Numbers (%)	Parental burnout *M* (P_25_, P_75_)
Gender		
Male	67(33%)	48(32,85)
Female	136(67%)	60(40,92.2)
*p*		0.015
Age		
< 30 year	50(24.6%)	64.5(44.7,93.5)
30 ~ 40 year	127(62.6%)	52(33,90)
> 40 year	26(12.8%)	48(35.7,70)
*p*		0.09
Monthly income (RMB)		
< 3,000	48(23.6%)	80.5(50.2,98.7)
3,000 ~ 6,000	94(46.4%)	57.5(36.7,90)
6,001 ~ 9,000	37(18.2%)	43(33,70)
> 9,000	24(11.8%)	41.5(31.5,56.7)
*p*		<0.001
Employment status		
Full-time job	93(45.8%)	60(36,86.5)
Part-time job	48(23.6%)	54.5(35.7,91.2)
Unemployed	62(30.5%)	55.5(33.2,87.2)
*p*		0.937
family structure		
Nuclear family	120(59.1%)	57.5(35,88.7)
Stem family	79(38.9%)	56(35,85)
single-parent family	4(2%)	47.5(37,85)
*p*		0.902
Gender of autistic children		
Male	157(77.3%)	56(35.5,84.5)
Female	46(22.7%)	59(34.7,92.2)
*p*		0.726
Age of autistic children		
≤ 3 year	30(14.8%)	64(35.7,95)
3 ~ 6 year	140(69%)	55(34,85)
≥ 6 year	33(16.3%)	55(37.5,96.5)
*p*		0.508
Degree of disease symptoms		
light	97(47.8%)	51(35,89)
moderate	85(41.9%)	55(33,77)
severe	21(10.3%)	68(42.5,102)
*p*		0.3
Duration of treatment		
Variable	Numbers *(%)*	Parental burnout *M (P_25_, P_75_)*
< 1 year	113(47.8%)	60(41,92.5)
1 ~ 3 years	88(41.9%)	47(33,70)
> 3 years	2(10.3%)	75(41,109)
*p*		0.056
Reimbursement method of treatment expenses		
Self-pay	22(10.8%)	89(75.2,95)
Medicare	82(40.4%)	55.5(39.7,87.2)
Business insurance	33(16.3%)	44(32.5,72.5)
Medicare and disability benefits	66(32.5%)	55(33,70)
*p*		0.007

### 3.2. Correlation analysis

Through correlation analysis, we found that the perceived social support was obviously and negatively correlated to alexithymia (*r* = −0.423, *p*<0.01) and parental burnout (*r* = −0.595, *p*<0.01). Besides, alexithymia was obviously and positively correlated to parental burnout (*r* = 0.674, *p* < 0.01; Table 2).

**Table 2:** Analysis of the correlation between alexithymia, perceived social support, and parenting burnout.

variable	Score (*X ± S*) or M *(P_25_, P_75_)*	1	2	3
1. Parental burnout	61(40,92.5)	1		
2. Alexithymia	58.90 ± 9.93	0.654^**^	1	
3. Perceived social support	52.36 ± 13.50	−0.372^**^	−0.513^**^	1

^**^p < 0.001.

### 3.3. Testing for the mediator

**Figure 1:**
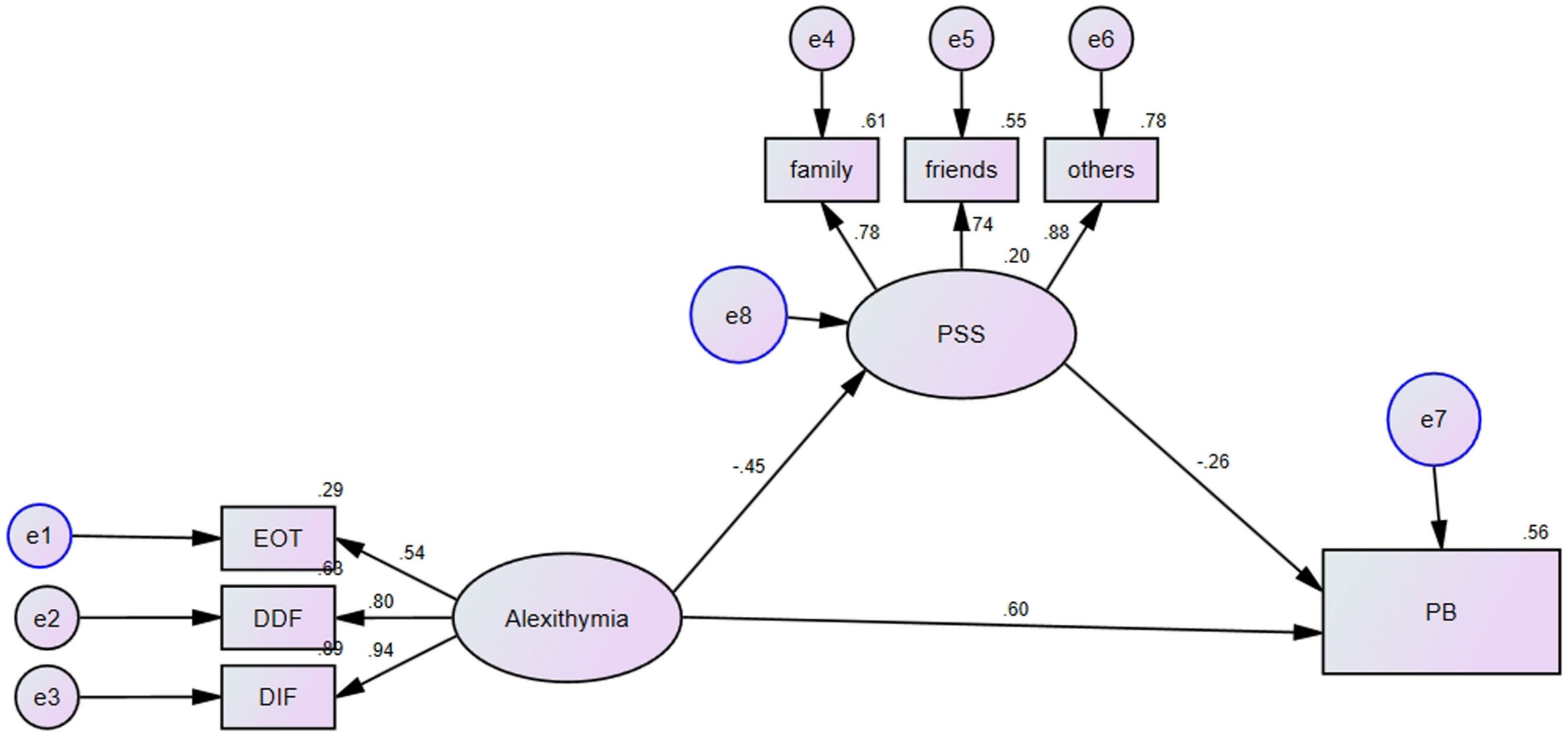
Structural equation model mediated by perceived social support. ^*^EOT, extremally oriented thinking; DDF, difficulty of describing feelings; DIF, difficulties with identifying feelings; PSS, perceived social support; PB, parental burnout.

Structural equation modeling was performed and AMOS was used to test the relationship between alexithymia, perceived social support, and parental burnout. Bootstrap testing and 95% CI range were used to test the significance of the indirect effects model. The final model had a good fit (*X*^2^/*df* = 1.3, GFI = 0.97, TLI = 0.99, CFI = 0.99, RMSEA = 0.03). The path coefficients of the structural equation model and the coefficient of direct and indirect effects are shown in Figure 1 and Table 3.

**Table 3:** Bootstrap test results of the mediation model.

	Point estimate	Product of coefficients		Bootstrapping			
				Bias-corrected 95% CI		Percentile 95% CI	
		SE	CR	Lower	Upper	Lower	Upper
Direct effect	A-PSS	−0.45	−5.126	−0.603	−0.256	−0.604	−0.257
	A-PB	0.6	10.45	0.477	0.699	0.478	0.699
	PSS-PB	−0.26	−3.8	−0.38	−0.113	−0.392	−0.126
Indirect effect	A-PSS-PB	0.116	3.31	0.6	0.208	0.05	0.191
Total effect	A-PBA	0.712	17.8	0.624	0.779	0.623	0.779

2,000 bootstrap samples.

We found that alexithymia can directly and positively affect parental burnout (*β* = 0.6, *p* < 0.01), so hypothesis 1 was supported. In addition, alexithymia negatively affected perceived social support (*β* = −0.45, *p* < 0.01). Perceived social support also negatively affected parental burnout (*β* = −0.26, *p* < 0.01). Alexithymia and perceived social support were able to explain 56% of the variance of parental burnout.

According to the results of the bootstrap test (Table 3), in the indirect pathway, the 95% CI range [0.05,0.191] did not include 0, indicating that perceived social support mediated the relationship between alexithymia and parental burnout. The total effect of alexithymia on parental burnout was 0.712, and the effect of alexithymia on parental burnout through perceived social support was 0.116 so the ratio of mediating to total effects was 16.3%.

### 3.4. Moderating effect test of gender

Based on the mediating model above, we conducted a multiple-group analysis using AMOS with males and females as group variables to explore the moderating role of gender. In the first model, we did not constrain any path coefficients (unconstrained model), the second model was a measurement–weighted model and the third model constrained all structural path coefficients of the male and female models to be equal (structural-weighted model). The results showed that all three models had good fitness (unconstrained structural model: *χ*^2^/*df* = 1.14, RMSEA = 0.03, CFI = 0.99, TLI = 0.99; measurement -weighted model: *χ*^2^/*df* = 1.28, RMSEA = 0.03, CFI = 0.98, TLI = 0.98; structural-weighted model: *χ*^2^/*df* = 1.57, RMSEA = 0.05, CFI = 0.97, TLI = 0.96).

**Figure 2:**
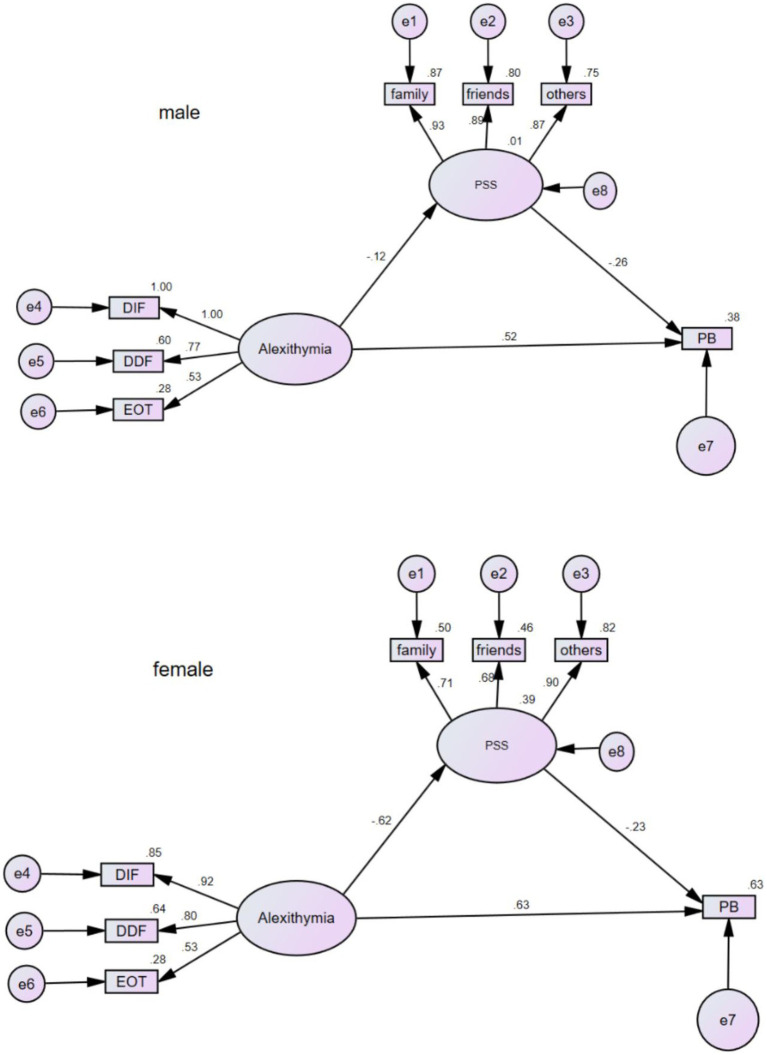
Multi-group structural equation model plot of gender difference.

The results showed that the chi-square difference between the measurement-weighted and unconstrained models was insignificant (Δ*χ*^2^ = 11.1, *p* = 0.086). The chi-square difference between the structural-weighted model and the unconstrained model was significant (Δ*χ*^2^ = 21.3, *p* = 0.003), indicating that mediated model differed by gender. This study further calculated the critical ratios of differences (CRD) to assess the between-group differences in each path coefficient. There was no gender difference between the two pathways from alexithymia to parental burnout (CRD = 1.074) and from perceived social support to parental burnout (CRD = –0.284). the path coefficients from alexithymia to perceived social support were significantly different (CRD = −2.554), indicating the moderating effect of gender on this pathway. The structural equation model plots for males and females are shown in Figure 2.

## 4. Discussion

Following a number of major sociological changes in recent decades, parenting methods have undergone profound changes, reflected in an increase in parental involvement and a more intensive approach to parenting (Roskam et al., 2021). Parental burnout has gradually attracted the attention of more and more researchers. Although many scholars have mentioned that parental burnout is affected by parental personality traits, only the effects of the Big Five personality traits on burnout have been studied (Le Vigouroux et al., 2017; Sekułowicz et al., 2022). There is little known about the impact of alexithymia, a separate personality trait, on parental burnout. Therefore, this study explores the relationship between alexithymia, perceived social support, and parental burnout in parents of autistic children. At the same time, we also explore the moderating role of gender in this.

Our study showed that the parental burnout score of parents of autistic children was 61(40,92.5), the higher monthly income and higher medical expense reimbursement contributed to the less sense of parental burnout. This finding clarified that rather than parents of typically developing children, parents of autistic children have experienced more nurturing environment burnout in China (Chen et al., 2022). The possible reason for this is that the development of pediatric rehabilitation in China is lagging behind, and the support facilities for disabled children are not yet complete, which means that parents cannot receive more rehabilitation support and face more parenting difficulties (Qi and Wang, 2021). In addition, because of the emphasis in Chinese culture on shame and ‘saving face’, some parents deny that their children have autism and refuse outside help, leading to a state of isolation and helplessness in parenting, and ultimately to high levels of parental burnout (Ji et al., 2022). As the same as the previous studies (Vigouroux and Scola, 2018), this study believes that economic condition is a vital factor in parental burnout. Adequate monthly income and commercial insurance that can cover treatment costs ensure that families receive more rehabilitation support, which can reduce the parenting burden. Therefore, to reduce parental burnout among parents of autistic children, the government is suggested to increase their welfare package and reimbursement level of treatment costs.

The equational model indicated that alexithymia had a strong positive effect on predicting parental burnout. People with a high level of alexithymia can feel greater psychological pressure and are more embedded to use evasive, self-blamed, and other invalid methods to cover with pressure, so they can feel more burnout (Wu et al., 2020; Li et al., 2022). Notably, the association between alexithymia and parental burnout is partially mediated by perceived social support. Due to the heritability of autism, some parents of autistic children exhibit subclinical features of autism, such as cognitive and communication deficits, and rigid and indifferent personality traits (The Broad Autism (Endo)Phenotype, n.d.), which have been shown to be associated with alexithymia (Leonardi et al., 2020). People with cognitive communication impairments are unable to understand and express emotions, which is an important factor in interacting socially. This makes it difficult for them to form intimate relationships with others (Shi et al., 2022). Reducing intimate relationships means reducing social support. Parents have less access to external resources in the parental environment, thereby increasing the likelihood of burnout. Therefore, as the result of this study show, parents of autistic children have high levels of alexithymia, and perceived social support plays a mediating role between alexithymia and parental burnout. It is suggested that medical personnel should pay attention to and solve the problem of alexithymia in parents of autistic children. Studies have shown that mindful meditation may reduce alexithymia by altering the perception of physical sensations and emotions (Aaron et al., 2020). Thus, health professionals are supported to develop mindfulness stress reduction courses to guide parents of autistic children. In addition, improving emotional expression can effectively change alexithymia. Therefore, cultivating parents’ emotional sensitivity through arts learning (Katsifaraki and Tucker, 2013; Cameron et al., 2014; such as reading, journaling, etc.) can also reduce alexithymia, thereby achieving the goal of reducing parental burnout.

It was found that mothers have stronger parental burnout than fathers, which is consistent with other studies (Mikolajczak and Roskam, 2018). Mothers often take on multiple roles in parenting activities. They are not only the daily caregivers of autistic children but also behavior educators (Parsons et al., 2017). Moreover, autistic children are less able to give positive emotional feedback to their parents in the process of childcare, so mothers may feel more tired of childcare. The results of the multi-group analysis showed that gender regulated the first half pathway in the mediation model. It means that women with alexithymia feel more sense of social isolation than men. This seems to provide some support for the “female vulnerability hypothesis,” which stated that women are more likely than men to develop psychological problems when they are exposed to stressful trauma events (Breslau et al., 1999). In addition, it may also be related to the fact that males avoid contact with their inner feelings and pay less attention to the degree of getting social support (Chung and Chen, 2020). In conclusion, this suggests that for mothers, alleviating alexithymia may improve their perceived social support and thereby reduce burnout. But for fathers, other methods are needed to be found to improve their perceived social support.

However, this study has some limitations that need to be acknowledged. Firstly, all the data in this study are from one city in China, which makes the samples lack representability. Next, some researchers mentioned that external measures should be used to measure the alexithymia of parents with autistic children, because they may have difficulty detecting their alexithymia symptoms. However, this study uses a self-assessment scale, which may affect the accuracy of the results. Finally, the disadvantage of cross-sectional studies is that causality cannot be determined. It means that perceived social support may also contribute to alexithymia, although theoretically, the proposed direction is feasible. Based on the limitations above, future studies can adopt multi-center surveys and include more cities to make the results more extrapolative. In addition, external measurement can be used to measure alexithymia, and longitudinal design can also be used in research design to provide a basis for more reliable results and causality.

## 5. Conclusion

Our research found that parents of autistic children in China have higher levels of burnout than the parents of typically developing children. Alexithymia of parents is significantly positively correlated with parental burnout and is moderated to some extent by perceived social support. Therefore, Chinese medical care staff should provide all aspects of support for families of autistic children, so as to reduce parental burnout.

## Data availability statement

The raw data supporting the conclusions of this article will be made available by the authors, without undue reservation.

## Ethics statement

The studies involving human participants were reviewed and approved by Ethics Committee of the Third Affiliated Hospital of Zhengzhou University. The patients/participants provided their written informed consent to participate in this study. Written informed consent was obtained from the individual(s) for the publication of any potentially identifiable images or data included in this article.

## Author contributions

YL, responsible for learning design and article writing. YW, responsible for data collection. CL, responsible for data collection. QN, RJ, and YQ, responsible for data analysis. YC, responsible for supervision. All authors contributed to the article and approved the submitted version.

## Funding

This work was supported by Henan Medical Scientific and technological breakthroughs Program Joint Construction Project (No. 2018020171).

## Conflict of interest

The authors declare that the research was conducted in the absence of any commercial or financial relationships that could be construed as a potential conflict of interest.

## Publisher’s note

All claims expressed in this article are solely those of the authors and do not necessarily represent those of their affiliated organizations, or those of the publisher, the editors and the reviewers. Any product that may be evaluated in this article, or claim that may be made by its manufacturer, is not guaranteed or endorsed by the publisher.
